# Bi-cultural dynamics for risk and protective factors for cardiometabolic health in an Alaska Native (Yup’ik) population

**DOI:** 10.1371/journal.pone.0183451

**Published:** 2017-11-01

**Authors:** Jacques Philip, Tove K. Ryman, Scarlett E. Hopkins, Diane M. O'Brien, Andrea Bersamin, Jeremy Pomeroy, Kenneth E. Thummel, Melissa A. Austin, Bert B. Boyer, Kirk Dombrowski

**Affiliations:** 1 Center for Alaska Native Health Research, University of Alaska Fairbanks, Fairbanks, Alaska, United States of America; 2 Bill and Melinda Gates Foundation, Seattle, Washington, United States of America; 3 Marshfield Clinic Research Institute, Marshfield, Wisconsin, United States of America; 4 Department of Pharmaceutics, University of Washington, Seattle, Washington, United States of America; 5 Department of Epidemiology, School of Public Health, University of Washington, Seattle, Washington, United States of America; 6 Department of Sociology, University of Nebraska, Lincoln, Nebraska, United States of America; Mayo Clinic Arizona, UNITED STATES

## Abstract

Alaska Native people experience disparities in mortality from heart disease and stroke. This work attempts to better understand the relationships between socioeconomic, behavioral, and cardiometabolic risk factors among Yup’ik people of southwestern Alaska, with a focus on the role of the socioeconomic, and cultural components. Using a cross-sectional sample of 486 Yup’ik adults, we fitted a Partial Least Squares Path Model (PLS-PM) to assess the associations between components, including demographic factors [age and gender], socioeconomic factors [education, economic status, Yup’ik culture, and Western culture], behavioral factors [diet, cigarette smoking and smokeless tobacco use, and physical activity], and cardiometabolic risk factors [adiposity, triglyceride-HDL and LDL lipids, glycemia, and blood pressure]. We found relatively mild associations of education and economic status with cardiometabolic risk factors, in contrast with studies in other populations. The socioeconomic factor and participation in Yup’ik culture had potentially protective associations with adiposity, triglyceride-HDL lipids, and blood pressure, whereas participation in Western culture had a protective association with blood pressure. We also found a moderating effect of participation in Western culture on the relationships between Yup’ik culture participation and both blood pressure and LDL lipids, indicating a potentially beneficial additional effect of bi-culturalism. Our results suggest that reinforcing protective effects of both Yup’ik and Western cultures could be useful for interventions aimed at reducing cardiometabolic health disparities.

## Introduction

Over the last three decades, attention to social determinants of health among indigenous communities have focused on individual and community social stressors that stem from the marginal place of indigenous communities in the current global political economy [1–5]. Initially this research focused on the discovery and examination of “risk factors” that influenced individual health outcomes [6–8]. More recently, researchers have also emphasized the importance of indigenous culture in influencing health outcomes [3,9–13]. The potential for local culture to act as protection against social harms and serve as a source of community resiliency, for example by promoting healthy traditional diets, represents what some have identified as significant change of research and intervention ideology [14].

Across the Arctic, culture-focused health interventions contend with a legacy of past, deleterious state-inspired programs such as community relocation and sedentarization [15–18]. These programs, originally intended to promote assimilation and the incorporation of Inuit/Inupiat/Yup’ik peoples in larger national political economies, have resulted in a well-known list of social ills, such as broken family ties or loss of language [19–21]. Emerging literature on ‘cultural-based protective factors’ in the region has focused on the potential for cultural participation to mitigate negative outcomes associated with this history [22], including suicide [23,24], substance abuse [19,25,26], Type 2 diabetes [27], depression [4,28] and other mental health outcomes [29,30].

Importantly, ethnographic (and other) research has shown that cultural protective factors are embedded in complex social and historical practices, in which protective and harmful effects may be intermingled [31–35]. In this view, culture is not a simple independent variable in the sense required for linear regression, nor is it monolithic and consistent in its effects on all individuals across a community or a region [36,37]. To fully enact culture-based interventions, a more complex understanding of the inter-relationships between culture and health behaviors among indigenous peoples is needed.

This study provides an analysis of the impact of cultural participation and other socioeconomic variables in relation to cardiometabolic risk factors within an Alaska Native population. Alaska Native people are more at risk of dying from heart disease or stroke [but not from diabetes] than non-Native people [38], justifying research aimed at addressing this disparity. Prior research with Alaska Native Yup’ik people established that cardiometabolic risk was associated positively with increased consumption of processed foods, and negatively with increased consumption of traditional foods [39]. Yet beyond the material aspect of nutrients brought by food, subsistence practices have a much more profound meaning for Yup’ik people, for example through norms around the respect for animals, rituals, and spirituality that may contribute to lasting socioeconomic relationships that carry indirect health benefits [40]. For example, the Yup’ik concept of Yuuyaraq, "The way of being a human being", defines how humans, animal, and nature interact in the Yup'ik way of life, which not only includes activities such as hunting, fishing, gathering, dances, and ceremonies, but also cultural values transmitted orally by Elders, such as sharing, humility, or conflict avoidance, and rules strongly tied to the spiritual world, like the correct way of speaking about living things, or how to handle hunted animals [41]. Traditional lifestyle was also found to be associated with higher levels of physical activity, lower perceived stress, and lower body mass index (BMI) [42], all potential counteractions to cardiometabolic risk.

In addition, a recent study showed that *iqmik* use (a traditional form of smokeless tobacco used by Yup’ik people) was negatively associated with cardiometabolic risk, and this association was attenuated after adjustment for BMI, suggesting a mediating effect of adiposity [43]. Here too cultural factors may be involved in this relationship; Wolsko et al [44] describe how *iqmik* is used by Yup’ik people as a way to cope with stress, which is often perceived as being induced by acculturation to a Western way of life [45]. In contrast, Boden-Albala et al [46] found that bicultural identity decreased the association between age and hypertension, and between education and hypertension, indicating that Yup’ik culture and Western culture can potentially interact in complex and potentially health-protective ways.

Operationalizing the concept of culture for statistical analysis offers significant challenges. In studies aimed at mobilizing cultural factors for health interventions, culture has been at times been defined as participation in activities linked to spirituality and ancient traditions—activities such as hunting, food gathering, the sharing of locally obtained foods, and traditional craft production based in these same activities— [29,47–49]. Others have used self-governance and local control associated to define cultural continuity [50–52]. Still others have looked at patterns of social exchange associated with kinship and indigenous/traditional knowledge as stand-ins for ongoing collective culture [37,53,54]. Here we follow a large number of researchers working in indigenous communities in the north and operationalize culture in both the community and individual sense through ongoing use of the indigenous language of the area and a self-assessment of “following a Yup’ik” lifestyle [28,29,55–57]. Research has shown that indigenous language use is often intrinsic to participation in traditional cultural activities [58,59], in effect capturing a range of health-related behaviors related to cardiometabolic risk. In addition, the term “traditional/Yup’ik lifestyle” is commonly used in Alaska as a signifier of participation in traditional, subsistence activities [36,60]. By looking culture as it has been defined and mobilized for health research by these researchers, we build on findings that demonstrate the potential efficacy of cultural participation for individual physical and mental health [58].

The analysis described below investigates the relationship among more than 20 socioeconomic, cultural, behavioral and cardiometabolic measures. To date we are not aware of studies that have attempted to evaluate such a wide range of potentially protective and deleterious factors in a single analytical framework. In this study, we seek to build on prior factor-specific findings by employing multivariate analytical techniques aimed at evaluating the complex interweaving of cultural, socioeconomic, behavioral and physical factors associated with both cardiometabolic risk and protection, while allowing culture (both Yup’ik and Western) to have interdependent and heterogeneous effects. The long-term goal of this research is to further define the interactions between culture and health so that they may be implemented in interventions aimed at improving the cardiometabolic health of Alaska Native people.

## Methods

The Center for Alaska Native Health Research (CANHR) was created in 2001 at the University of Alaska Fairbanks, with the primary aim to identify behavioral, genetic, and nutritional risk and protective factors contributing to obesity and Type 2 diabetes (T2D) in Yup’ik Alaska Native people residing in Southwest Alaska. Detailed descriptions of study design and recruitment methods have been previously published [61,62]. The majority ethnic identity in the region is Yup’ik, with a minority identifying as Cup’ik, with both groups falling broadly under Yup’ik culture.

### Participants

The analytic sample for this article includes participants recruited from 9 rural communities in the Yukon-Kuskokwim Delta with populations between 250 and 700, plus a hub community of ~6,000 residents. Between September 2009 and May 2013, CANHR investigators made recurrent visits to each community. All community members 14 years or older, self-identified as Alaska Native descent or married to an Alaska Native individual, and non-pregnant were invited to participate. All eligible participants were enrolled at each visit and thus the data consist of multiple cross-sectional, convenience samples. For this analysis, participants include adults (≥18 years of age), who self-identified as either Yup’ik or Cup’ik, who had completed measures for cardiometabolic risk factors, dietary and physical activity assessment, and had been fasting. For participants with multiple visits, the latest one was used. From this sample of 620, an additional 134 participants were excluded because they were on diabetes, hypertension, or dyslipidemia control medication, as a way to reduce the bias related to the fact that their cardio-metabolic risk factors are artificially lowered, bringing the final sample to 486 individuals.

All procedures involving study participants were approved by the University of Alaska Fairbanks Institutional Review Board and the Yukon-Kuskokwim Health Corporation Human Studies Committee. Written informed consent was obtained in English or Yup’ik from participants prior to data collection.

### Measures

At each visit, participants answered questionnaires in English or Yup’ik. These included demographic characteristics, socioeconomic and cultural questions, medical history, medication usage, fasting status, tobacco use behavior, and diet from food frequency questionnaire (FFQ). Weight, height and other anthropometric measurements were collected during a physical exam by trained investigators. Physical activity was measured by an Actiheart system, monitoring movement and heart rate.

#### Socio-economic and cultural measures

The assessment of education, economic status, and culture participation is described in Table 1. The two lifestyle questions were developed by CANHR researchers following focus groups and interviews of Yup’ik participants’ conceptualizations of health and wellness [45,63] and based on the orthogonal model of cultural identification, which posits that identification patterns with different cultures are independent of each other, rather than being along a linear continuum [64].

**Table 1:** Variables, questions and options for socioeconomic and cultural measures.

Variables	Questions	Options/Scale
**Education**		
Number of school years completed^a^	*What is the last grade of school (K-12) you completed*?	0–12
	*How many years of college did you complete*?	Number of years
**Economic status**		
Runs out of money for food	*How often does your money for household utilities (ie water*, *fuel oil*, *electricity*, *etc) run out before the end of the month*?	1:Never, 2:Seldom, 3:Sometimes, 4:Most times, 5:Always
Runs out of money for utilities	*How often does your money for food run out before the end of the month*?	1:Never, 2:Seldom, 3:Sometimes, 4:Most times, 5:Always
**Western Culture**		
Main language spoken at home is English^b^	*Which language do you speak most often at home*	Cup’ik, Yup’ik, English, Other
Follows Western lifestyle	*How much do you follow the White (or Kass'aq) way of life*	1:Not at all, 2:Some, 3:A lot
**Yup'ik Culture**		
Speaks Yup’ik or Cup’ik^c^^d^	*Which languages do you speak*?	Cup’ik, Yup’ik, English, Other
Main language spoken at home is Yup’ik or Cup’ik^d^	*Which language do you speak most often at home*	Cup’ik, Yup’ik, English, Other
Follows Yup’ik lifestyle	*How much do you follow the traditional Yup'ik way of life*	1:Not at all, 2:Some, 3:A lot

^a^The score is the sum of scores of the two question.

^b^The score is 2 if the answer is English, 1 otherwise.

^c^Multiple answers allowed.

^d^The score is 2 if the answer includes Yup’ik or Cup’ik, 1 otherwise.

#### Lifestyle measures

Dietary intake was assessed with both self-reported FFQ and objectively measured stable isotope biomarkers. A culturally specific Yup’ik FFQ was developed and validated by CANHR [65,66]. The FFQ data were used to identify three dietary patterns of traditional or subsistence foods (e.g., walrus or seal), processed foods, and fruits and vegetables. The dietary patterns were used to calculate dietary pattern scores representing the relative frequency of consumption for each. Details of the dietary patterns have been published previously [65,66].

Stable isotope ratios were used as dietary biomarkers for fish and marine mammal intake and processed food intake. In this population, the nitrogen isotope ratio (^15^N/^14^N, expressed as the δ^15^N value) is strongly associated with fish and marine mammal intake [67–69]. The carbon isotope ratio (^13^C/^12^C or the δ^13^C value) is elevated in corn and sugar-based foods, and is associated with processed food intake when assessed via FFQ [66].

Physical activity was measured by asking participants to wear a combined heart rate/movement device (Actiheart, CamNtech Ltd, Papworth UK), for four consecutive days. We used the average daily total activity counts as a measure of all physical activity regardless of intensity, the moderate to vigorous activity time classified as time where heart rate was at least 1.75 times resting heart rate, and sedentary time which was classified as minutes with less than 10 accelerometry counts per minute and a valid heart rate less than 1.10 times resting heart rate (rev. coded). All three measures were adjusted for actual wear time of the device, which varied between 10 and 24 hours per day.

For both cigarette smoking and smokeless tobacco use, a categorical measure including the levels of current, past, and never user was defined, with current use representing any use at the time of interview, regardless of the amount. We also defined a continuous variable as the number of cigarettes or chews per day currently used. Smokeless tobacco use among Yup’ik people in this study includes mainly *Iqmik*, a homemade mixture of cured tobacco leaves and ash from *Phellinus igniarius*, a fungus commonly found on birch and alders, or ash from willow or alder [70]. Smokeless tobacco also included to a much smaller extent commercial chew and snuff [44].

#### Cardiometabolic risk factors

Blood was drawn after 12 hours of fasting and processed locally into plasma, serum, buffy coat and red blood cells (RBC). Blood samples were analyzed locally for fasting plasma glucose (FPG) and HbA1C and plasma was sent for analysis to UC Davis University for lipids. Resting systolic and diastolic blood pressure were measured on participants, using the OMRON HEM907 automated blood pressure cuff. The mean of the last 2 out of three measures was used for analysis. Waist circumference (WC) was measured twice using a Gulick II 150 cm anthropometric tape attached with a tension-meter immediately below the lowest lateral portion of the rib case. The average of two measures was used for analysis. BMI was calculated as weight (kg)/height (m2). More details on these protocols have been published previously [39].

### Analysis

We adopted a two-step approach for our analysis, with (1) a Principal Components Analysis (PCA) of the cardiometabolic risk factors to reduce those nine variables into fewer components, and (2) a Partial Least Square Path Model (PLS-PM) to explore how demographic, socioeconomic, and behavioral factors predict those risk factors components. PLS-PM is a non-parametric alternative method to structural equation modeling (SEM), commonly used in econometrics [71], which allows for the construction of a path model between a set of latent variables (inner model), each of which being represented by a block of correlated measured or manifest variables (outer model). Compared to SEM, PLS-PM does not require normally distributed variables, allows easier convergence for small samples relative to the number of variables and cases, and is more adapted for exploring a model than strict testing of strongly established hypothesis [72–74]. The disadvantage over SEM is that PLS-PM produces less precise estimates [75].

All analyses were conducted with the R software, v. 3.2 [76], using the Psych package for PCA, and the plspm package for PLS-PM analysis. Missing values were replaced by the median for that variable across participants.

#### Principal Component Analysis

We based our PCA of cardiometabolic risk factors on the idea proposed by several authors that continuous scores of components of the metabolic syndrome (MetS) have better predictive power than its dichotomous standard definition [77–79]. Using commonly accepted criteria defining the MetS, i.e., abdominal obesity, an index of insulin resistance/glucose intolerance, dyslipidemia, and hypertension [80,81], We conducted the PCA on the following variables: Waist Circumference, BMI, LDL-C, - HDL-C (reverse coded), Triglycerides, Systolic BP, Diastolic BP, Glucose, and HbA1C, requiring five components with varimax rotation. Even though our PCA is based on definitions of the MetS, the resulting components do not represent a measure of the MetS, but simply five continuous dimensions of cardiometabolic risk.

#### Partial Least Square Path analysis

**Fig 1:**
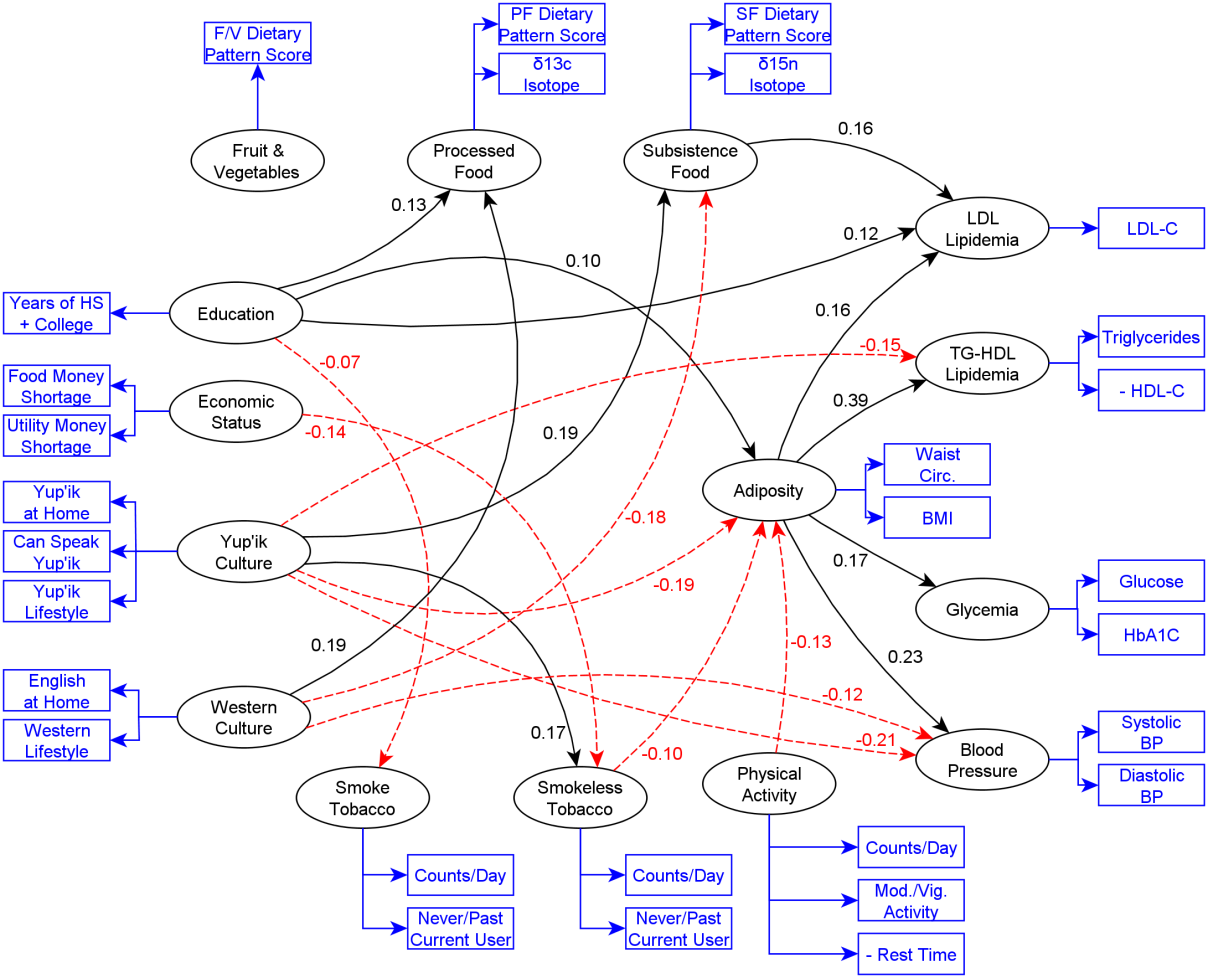
PLS-PM relationships with total effect coefficients between all latent variables other than gender and age. Significant effects only (α = 0.05). Ellipses are latent variables; rectangles are measured variables composing each latent variable. Plain black lines and red dotted lines represent positive and negative associations respectively.

A PLS-PM model is defined by an outer and an inner model, as represented by Fig 1. To avoid repeating a very similar figure, Fig 1 is used to both show the topology of the model here, and the significant paths later in the results. In the outer or measurement model each latent variable or component is defined as a weighted linear combination of its manifest variables. For example, the latent variable Yup’ik culture participation is defined by its manifest variables Yup’ik at home, Speaks Yup’ik, and Yup’ik lifestyle. The inner or structural model defines the relationships between latent variables. Those relationships are evaluated as a set of linear regressions, one for each endogenous latent variable (a latent variable that has at least one incoming path). In each regression, that endogenous variable is predicted by all variables that have a path to it. For example, adiposity is predicted by behavioral, socioeconomic, and demographic components. The path coefficients are those regression coefficients and are labeled as effects, in PLS-PM terminology, but should be understood as associations, since our analysis uses PLS-PM in an exploratory fashion. These regressions are fitted iteratively as the weights of the outer model are optimized; convergence is obtained when the residual overall variance is minimized. Confidence intervals can be estimated by non-parametric methods [73,82]. Here we used 1000 bootstrapping repetitions, and confidence level was set at 0.05. We did not use multiple comparison adjustment because we are not formally testing hypothesis, but we are using PLS-PM in an exploratory fashion.

After the components of the cardiometabolic risk factors were identified, we included them in the inner model, i.e., demographic components [Gender and Age], socioeconomic components [Education, Economic Status, Western Culture, and Yup’ik Culture], and behavioral components [traditional diet, processed diet, and fruits and vegetables diet, physical activity, cigarette smoking, and smokeless tobacco use]. We then established directional relationships between components that are theorized to predict others. Thus we defined a path between each demographic and behavioral components and cardiometabolic risk factors components. In addition, we defined a path from adiposity to each of the other cardiometabolic risk factors components given the central role of adiposity the International Diabetes Federation (IDF) definition of the metabolic syndrome [81]. Downstream from the cardiometabolic risk factors, we defined paths from each of the socioeconomic components to the behavioral components and from the demographic components to the behavioral components as well as the socioeconomic components.

We also defined a model which includes an interaction between Western and Yup’ik cultures, based on previous research suggesting that bi-culturalism may have a protective effect on hypertension [46]. Among the different methods to compute an interaction effect in PLS-PM, we elected to use the two stage approach where the first stage consists of fitting the model without interaction as previously described, and the second of fitting an interaction model, which adds an interaction latent variable made of the score of Western and Yup’ik cultures obtained from the first model [83]. The interaction effect can then be assessed by coefficients of the paths between the interaction and the other latent [outcome] variables, and visualized by using the model to predict each outcome variable for each level of Western and Yup’ik cultures.

## Results

Participant characteristics are presented in Table 2. In summary, 51% of participants were female and the average age ± sd was 35.8 ± 15.4 years. The average education level was slightly below high school graduation at 11.3 ± 1.9 years. Our two economic status variables are relatively balanced around the median. 95.1% of participants speak Yup’ik or Cup’ik and the majority (69%) of participants speak mostly Yup’ik or Cup’ik at home. Participants speaking mostly English are almost the reciprocal except for one who reported speaking neither. Almost all participants report following the Yup’ik lifestyle “some” or “a lot” (50% and 49%, respectively), and most follow the western lifestyle “some” or “a lot” (77% and 20%, respectively). Percentages of never, past and current tobacco users are respectively 34, 32, and 34 for cigarettes, and 16, 10, and 74 for smokeless tobacco. The overall average number of cigarettes smoked per day among current smokers is 4.7 ± 4.3. The overall average number of chews or snuffs per day among current users is 4.9 ± 3.9. Dietary intake, activity and risk factor variables had similar distributions than was published previously for the same population [43].

**Table 2:** Descriptive statistics of variables.

	n (%)	Mean ± sd	Skew
**Gender**			
Male	238 (49.0)		
Female	248 (51.0)		
**Age (yrs)**			
Continuous	486	35.8 ± 15.4	0.7
**Number of school years completed (HS + college) (yrs)**			
Continuous	486	11.3 ± 1.9	-1.2
**Runs out of money for food** ^b^			
Never	41 (8.4)		
Seldom	37 (7.6)		
Sometimes	159 (32.7)		
Most times	107 (22.0)		
Always	80 (16.5)		
Missing	62 (12.8)		
**Runs out of money for utilities** ^b^			
Never	90 (18.5)		
Seldom	61 (12.6)		
Sometimes	132 (27.2)		
Most times	59 (12.1)		
Always	77 (15.8)		
Missing	67 (13.8)		
**Main language spoken at home is English**			
No	338 (69.5)		
Yes	148 (30.5)		
**Follows Western lifestyle**			
Not at all	15 (3.1)		
Some	375 (77.2)		
A lot	95 (19.5)		
Missing	1 (0.2)		
**Main language spoken at home is Yup’ik or Cup’ik**			
No	149 (30.7)		
Yes	337 (69.3)		
**Follows Yup’ik lifestyle**			
Not at all	5 (1.0)		
Some	242 (49.8)		
A lot	239 (49.2)		
**Speaks Yup’ik or Cup’ik**			
No	24 (4.9)		
Yes	462 (95.1)		
**δ15N isotope level (**‰**)**			
Continuous	486	8.8 ± 1.3	0.8
**Subsistence food dietary pattern score**^a^			
Continuous	486	0.2 ± 2.3	-0.3
**δ13C isotope level (**‰**)**			
Continuous	486	-19.7 ± 0.7	-0.4
**Processed foods dietary pattern score**^a^			
Continuous	486	0.8 ± 2.2	-0.4
**Fruits and vegetables dietary pattern score**^a^			
Continuous	486	-0.0 ± 2.2	-0.3
**Activity counts per day (count)**			
Continuous	486	60767.1 ± 37769.1	1.9
**Moderate to vigorous activity time per day (minutes)**			
Continuous	486	201.9 ± 176.9	1.4
**Rest time per day (minutes)**^b^			
Continuous	486	940.3 ± 163.7	-1.5
**Never, past or current smoke tobacco user**			
Never Smoker	166 (34.2)		
Past Smoker	153 (31.5)		
Current Smoker	167 (34.4)		
**Cigarettes smoked per day (count)**			
Continuous	486	1.6 ± 3.4	3.3
Among smokers		4.7 ± 4.3	
**Never, past or current smokeless user**			
Never Smokeless	79 (16.3)		
Past Smokeless	47 (9.7)		
Current Smokeless	360 (74.1)		
**Smokeless tobacco uses per day (count)**			
Continuous	486	3.6 ± 3.5	1.3
Among users		4.9 ± 3.9	
**Waist circumference (cm)**			
Continuous	486	87.6 ± 14.1	1
**Body Mass Index (kg/m**^**2**^**)**			
Continuous	486	26.7 ± 5.7	1.1
**Plasmatic HDL cholesterol (mg/Dl)** ^b^			
Continuous	486	62.9 ± 18.1	0.9
**Plasmatic triglycerides (mg/Dl)**			
Continuous	486	82.4 ± 44.3	3.2
**Fasting plasmatic glucose (mg/Dl)**			
Continuous	486	90.8 ± 9.8	0.3
**Hemoglobin A1C (%)**			
Continuous	486	5.5 ± 0.3	0.5
**Systolic blood pressure (mm Hg)**			
Continuous	486	116.3 ± 13.4	0.7
**Diastolic blood pressure (mm Hg)**			
Continuous	486	68.0 ± 9.5	0.3
**Plasmatic LDL cholesterol (mg/Dl)**			
Continuous	486	124.7 ± 34.8	0.7

^a^Pattern scores are calculated from food frequencies [65]

^b^Variable is reverse coded

### Principal Component Analysis

Table 3 shows the variance partition and variable loadings of the five components of the PCA of cardiometabolic risk factors. Adiposity explains 23% of the overall variance, blood pressure 19%, LDL lipids 15%, glycemia 14%, and TG-HDL lipids 14%, for a total of 84% of the total variance. The variables with highest loadings were retained for each component, to be used subsequently in the PLS-PM analysis as follows: waist circumference and BMI for adiposity, systolic and diastolic measures for blood pressure, just LDL-C for LDL lipids, glucose and HbA1C for glycemia, and—HDL-C and triglycerides for TG-HDL lipids.

**Table 3:** Components of the metabolic syndrome.

	Adiposity	Blood Pressure	LDL Lipids	Glycemia	TG-HDL Lipids
**Variance partition**					
SS loadings	2.03	1.67	1.34	1.27	1.24
Proportion Var	0.23	0.19	0.15	0.14	0.14
Cumulative Var	0.23	0.41	0.56	0.70	0.84
**Loadings**					
Waist Circ.	**0.93**	0.14	0.06	0.15	0.18
BMI	**0.96**	0.09	0.04	0.10	0.13
LDL-C	0.19	0.05	**0.83**	0.08	0.27
- HDL-C	0.21	-0.06	-0.66	0.04	**0.49**
Triglycerides	0.20	0.10	0.07	0.10	**0.87**
Systolic BP	-0.03	**0.93**	0.06	0.04	-0.02
Diastolic BP	0.28	**0.87**	0.04	0.02	0.14
Glucose	0.11	0.01	-0.12	**0.86**	0.25
HbA1C	0.17	0.07	0.44	**0.69**	-0.19

Five components explain 84% of the variability of 9 variables in a PCA analysis.

### PLS-PM analysis

#### Main effects

Table 4 displays outer model fitting characteristics which were marginal to good for most variables, after we removed other variables (not showed), because they altered unidimentionality or overall goodness of fit. For example loadings which should ideally be close to or above 0.7, were 0.8, 0.7 and 0.64 for the Yup’ik culture variables (*Yup’ik at Home*, *Yup’ik lifestyle* and *Can speak Yup’ik*, respectively). The variables *Mod*.*/vig*. *Activity*, *Triglycerides*, and *Glucose* had the lowest loadings. Sensitivity analysis without those variables did not show significant differences in the results or overall goodness of fit. Additionally, crossloadings, which evaluate if each manifest variable always loads higher for its theorized latent variable that for the others, were calculated and were adequate, but are not presented in the table. Overall the fitting of the outer model is acceptable.

**Table 4:** Weights, loadings, communalities and unidimensionality for outer Partial Least Squares Path Model (n = 486).

Variables	weight	loading	communality	C.alpha	DG.rho	eig.1st	eig.2nd
**Gender**				1.000	1.000	1.000	0.000
Gender	1.000	1.000	1.000				
**Age**					1.000	1.000	1.000
Age	1.000	1.000	1.000				
**Education**				1.000	1.000	1.000	0.000
Years of HS + college	1.000	1.000	1.000				
**Economic status**				0.732	0.882	1.577	0.423
Food money shortage (rev)	0.592	0.900	0.810				
Utility money shortage (rev)	0.534	0.875	0.766				
**Western culture**				0.481	0.794	1.317	0.683
English at home	0.822	0.938	0.879				
Western lifestyle	0.366	0.627	0.393				
**Yup’ik culture**				0.517	0.757	1.532	0.832
Yup’ik at Home	0.536	0.795	0.632				
Yup’ik lifestyle	0.482	0.693	0.481				
Can speak Yup’ik	0.374	0.642	0.412				
**Subsistence food**				0.483	0.795	1.318	0.682
δ15N	0.847	0.951	0.904				
SF dietary pattern	0.327	0.596	0.355				
**Processed food**				0.418	0.775	1.264	0.736
δ13C	0.461	0.666	0.443				
PF dietary pattern score	0.774	0.896	0.802				
**Fruits and vegetables**				1.000	1.000	1.000	0.000
FV dietary pattern score	1.000	1.000	1.000				
**Physical activity**				0.544	0.763	1.838	1.008
Counts/day	0.526	0.867	0.752				
Mod./vig. activity	0.497	0.513	0.263				
Rest time (rev)	0.368	0.784	0.615				
**Smoke tobacco**				0.735	0.883	1.581	0.419
Never/past/current user	0.685	0.936	0.876				
Counts/day	0.432	0.830	0.689				
**Smokeless tobacco**				0.740	0.885	1.587	0.413
Never/past/current user	0.465	0.849	0.720				
Counts/day	0.653	0.927	0.858				
**Adiposity**				0.959	0.980	1.921	0.079
Waist circ.	0.517	0.980	0.961				
BMI	0.504	0.979	0.959				
**TG-HDL Lipids**				0.432	0.779	1.275	0.725
HDL-C (rev)	0.937	0.985	0.971				
Triglycerides	0.177	0.434	0.189				
**Glycemia**				0.469	0.790	1.307	0.693
Glucose	0.109	0.404	0.163				
HbA1C	0.961	0.995	0.989				
**Blood Pressure**				0.802	0.910	1.669	0.331
Systolic BP	0.613	0.934	0.873				
Diastolic BP	0.480	0.890	0.793				
**LDL Lipids**				1.000	1.000	1.000	0.000
LDL-C	1.000	1.000	1.000				

*Note*. Weight = relative contribution of each manifest variable to the aligned latent variable; outer model fitting characteristics (recommended values) are: Loading = association of each manifest variable and the aligned latent variable (≥ 0.7); Communality = squared loadings representing proportion of variance in each manifest variable captured by the aligned latent variable (≥ 0.5); α = Cronbach's *alpha* (>0.7); ρ = Dillon-Goldstein's *rho* (>0.7); 1Eig = 1^st^ eigenvalue (>1); 2Eig = 2^nd^ eigenvalue (recommended < 1. Variables labels in **bold** are latent variables; variables labels in plain text are manifest variables.

Table 5 presents the inner model’s coefficients and 95% CI or p < .05 for significant total effects. A coefficient corresponds to a normalized regression slope and is considered statistically significant if its CI does not contain 0. A construct’s total effect is the sum of its direct and indirect effects, the latter being obtained by multiplying the paths coefficients of indirect pathways.

**Table 5:** Total significant effects coefficients, for inner Partial Least Squares Path Model, with Bootstrap Standard Error and 95% confidence intervals (n = 486).

Path	Coeff.	CI		Path	Coeff.	CI	
Gender -> Physical activity	-0.202*	-0.285	-0.121	Education -> Smoke tobacco	-0.069*	-0.138	0.000
Gender -> Smoke tobacco	-0.350*	-0.422	-0.274	Education -> Adiposity	0.100*	0.023	0.184
Gender -> Smokeless tobacco	0.137*	0.048	0.227	Education -> LDL Lipids	0.124*	0.025	0.215
Gender -> Adiposity	0.134*	0.052	0.220	Economic status -> Smokeless tobacco	-0.136*	-0.225	-0.045
Gender -> TG-HDL Lipids	-0.245*	-0.318	-0.174	Western culture -> Subsistence food	-0.175*	-0.260	-0.077
Gender -> Blood Pressure	-0.272*	-0.357	-0.182	Western culture -> Processed food	0.185*	0.066	0.289
Age -> Economic status	-0.169*	-0.264	-0.074	Western culture -> Blood Pressure	-0.120*	-0.241	-0.001
Age -> Western culture	-0.216*	-0.294	-0.135	Yup’ik culture -> Subsistence food	0.185*	0.081	0.294
Age -> Yup’ik culture	0.318*	0.239	0.401	Yup’ik culture -> Smokeless tobacco	0.170*	0.030	0.322
Age -> Subsistence food	0.492*	0.422	0.564	Yup’ik culture -> Adiposity	-0.188*	-0.335	-0.026
Age -> Processed food	-0.513*	-0.574	-0.434	Yup’ik culture -> TG-HDL Lipids	-0.145*	-0.256	-0.036
Age -> Fruits and vegetables	-0.198*	-0.285	-0.112	Yup’ik culture -> Blood Pressure	-0.205*	-0.332	-0.075
Age -> Physical activity	-0.320*	-0.397	-0.234	Subsistence food -> LDL Lipids	0.159*	0.056	0.259
Age -> Smoke tobacco	-0.185*	-0.267	-0.110	Physical activity -> Adiposity	-0.133*	-0.211	-0.050
Age -> Smokeless tobacco	0.116*	0.015	0.199	Smokeless tobacco -> Adiposity	-0.103*	-0.187	-0.018
Age -> Adiposity	0.202*	0.121	0.288	Adiposity -> TG-HDL Lipids	0.393*	0.323	0.465
Age -> TG-HDL Lipids	-0.413*	-0.475	-0.333	Adiposity -> Glycemia	0.169*	0.075	0.275
Age -> Glycemia	0.469*	0.375	0.539	Adiposity -> Blood Pressure	0.232*	0.130	0.330
Age -> Blood Pressure	0.311*	0.230	0.391	Adiposity -> LDL Lipids	0.159*	0.066	0.268
Age -> LDL Lipids	0.469*	0.402	0.539				

* Significant effects (α = 0.05)

**Fig 2:**
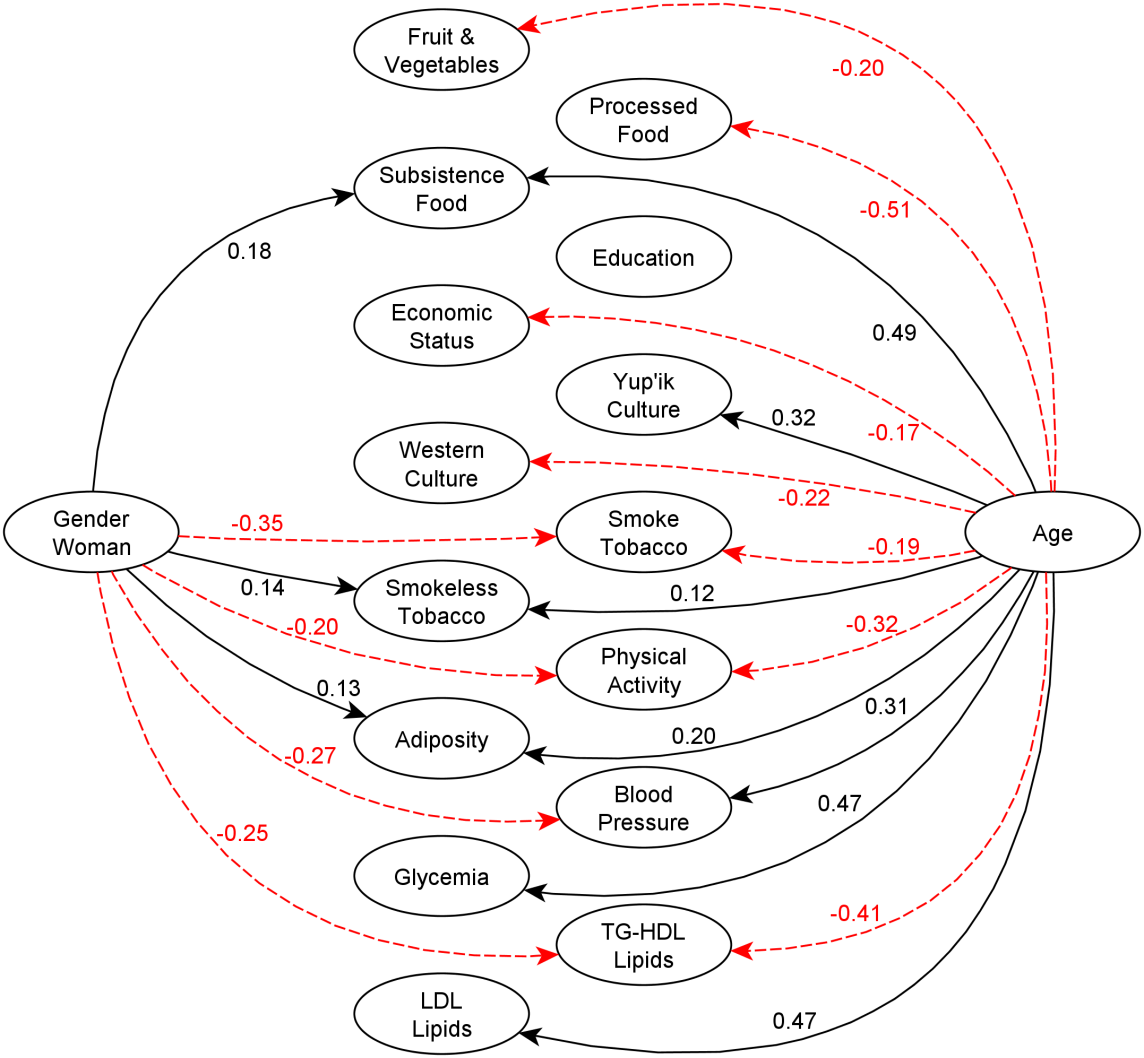
PLS-PM relationships with total effect coefficients of gender and age with all other latent variables. Significant effects only (α = 0.05). Note that Fig 1 and Fig 2 represent the same model and are separated only for ease of viewing. Plain black lines and red dotted line represent positive and negative associations respectively.

For clarity, the total effects in the PLS-PM analysis inner and structural model are split into Figs 1 and 2, and non-significant relationships have been omitted. Fig 1 shows the relationships between the socioeconomic, behavioral and cardiometabolic risk components; the behavioral to cardiometabolic risk components; and adiposity to other cardiometabolic risk components. Among these variables, the strongest associations were Adiposity -> TG-HDL Lipids (0.39), Adiposity -> Blood Pressure (0.23), Yup’ik culture -> Blood Pressure (-0.21), Yup’ik Culture -> Adiposity (-0.19), Western Culture -> Processed food, and Yup’ik Culture -> Subsistence Food (0.19). Fig 2 shows the relationships of age and gender to all other components. Here we see that age had the strongest associations (coefficient size) with other latent variables, e.g., with Processed Food (-0.51), Subsistence Food (0.49), LDL lipids (0.47), and Glycemia (0.46) respectively. Gender also had a strong reverse association with Smoke Tobacco (-0.35).

The results of the model show that (1) education had positive associations with processed food, adiposity, and LDL-lipids, and an inverse association with tobacco smoking; (2) economic status had an inverse association with smokeless tobacco use; (3) Yup’ik culture had positive associations with subsistence food and smokeless tobacco, and inverse associations with TG-HDL Lipids, adiposity, and blood pressure; and (4) Western culture had a positive association with processed food, and negative associations with subsistence food and blood pressure.

We also found significant paths afferent to some of the cardiometabolic risk components: (1) subsistence food had a positive association with LDL lipids; (2) smokeless tobacco and physical activity had inverse associations with adiposity; and (3) adiposity had positive associations with all other cardiometabolic risk components.

The overall goodness-of-fit criterion (0 < GoF < 1; (Tenenhaus et al., 2005) for this model is calculated as the geometric mean of the average communality and the average R2 value, and represents the average predictive power of the model. GoF = .35 barely falls short of the cut-off value of .36 proposed by Wetzels et al. [84] for a large effect size, but also indicates a substantial proportion of variability not captured by the model.

The complete direct path and total effect coefficients (including non-significant relationships), their standard errors, and 95% confidence intervals or p < .05 level are available in the Table in S1 Table.

#### Interaction effects

**Fig 3:**
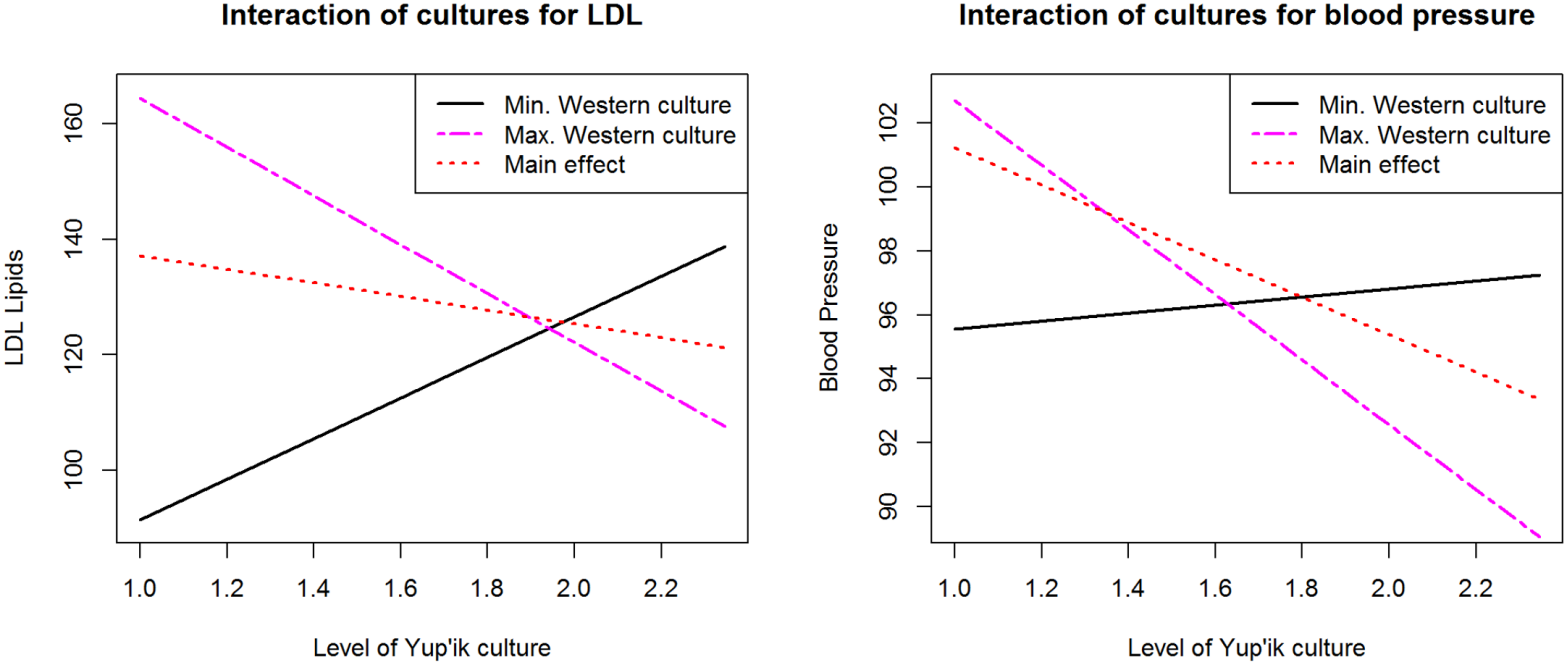
Model predictions of LDL and blood pressure for Western and Yup’ik cultures interaction by level of Yup’ik culture. Black full line represents the effect of Yup’ik culture for the lowest level of Western culture; purple dashed line represents the effect of Yup’ik culture for the highest level of Western culture; Red dotted line represents the main effect.

Table 6 presents coefficient, SE, and 95% CI of the direct paths and total effects for the interaction of Yup’ik and Western cultures. Fig 3 visualizes the interaction for the two variables where the interaction had a significant total effect. Each plot represents the predicted level of the dependent variable, LDL Lipids or Blood Pressure, as a function of the Yup’ik cultural participation level for the minimum and maximum Western cultural participation levels. We see that for the lowest Western culture level, there is a positive association of Yup’ik culture with LDL Lipids (the slope for the black continuous line increases showing that greater Yup’ik cultural participation predicts greater relative LDL Lipids levels), whereas for the highest level of Western cultural participation, the association is inverted (the slope for the red dashed line is negative, showing that greater Yup’ik cultural participation predicts lower relative LDL Lipid levels). Below we discuss this effect as indicating a qualified but significant protective effect of bi-culturalism. The main effect shows a slightly negative association between the two. We observe a similar result for blood pressure. Here the positive slope for lowest level of Western culture is not as steep as the one of LDL Lipids, indicating a weaker association between Yup’ik cultural participation and blood pressure than with LDL Lipids for lowest levels of Western culture.

**Table 6:** Direct paths and total effects coefficients of interaction between Western and Yup’ik culture, for inner Partial Least Squares Path Model, with Bootstrap Standard Error and 95% confidence intervals (n = 486).

Path	Direct Path				Total Effect			
	Coefficient	Std.Error	CI low	CI High	Coefficient	Std.Error	CI low	CI High
Culture interaction -> Subsistence food	-0.038	0.047	-0.126	0.054	-0.038	0.047	-0.126	0.054
Culture interaction -> Processed food	0.047	0.053	-0.058	0.151	0.047	0.053	-0.058	0.151
Culture interaction -> Fruits and vegetables	-0.013	0.065	-0.132	0.119	-0.013	0.065	-0.132	0.119
Culture interaction -> Physical activity	-0.053	0.071	-0.194	0.084	-0.053	0.071	-0.194	0.084
Culture interaction -> Smoke tobacco	0.060	0.062	-0.067	0.176	0.060	0.062	-0.067	0.176
Culture interaction -> Smokeless tobacco	0.020	0.065	-0.097	0.156	0.020	0.065	-0.097	0.156
Culture interaction -> Adiposity	-0.118	0.068	-0.255	0.018	-0.114	0.068	-0.249	0.016
Culture interaction -> TG-HDL Lipids	-0.054	0.048	-0.151	0.044	-0.092	0.052	-0.194	0.012
Culture interaction -> Glycemia	0.006	0.056	-0.097	0.113	-0.019	0.058	-0.128	0.091
Culture interaction -> Blood Pressure	-0.085	0.055	-0.188	0.022	-0.112*	0.055	-0.222	-0.003
Culture interaction -> LDL Lipids	-0.183*	0.060	-0.296	-0.061	-0.211*	0.060	-0.327	-0.089

* Significant effects (α = 0.05)

## Discussion

Most of these results point to complex associations between culture, behavior, and cardiometabolic health. The associations of education (often thought as a source of assimilation [85]) with processed food consumption, adiposity and LDL lipids suggests a potentially deleterious effect of western cultural participation, for example by exposing students to processed food lunches. However the absence of a significant path from processed food to LDL lipids suggests that either our processed food latent variable does not capture the specific processed foods that increase LDL-C, or that education is associated with LDL lipids through another unmeasured mechanism. It is also possible that more educated participants are more likely to have office jobs and have no time to obtain subsistence foods as we adjusted for income. The reverse association of education with smoked tobacco however may represent possible protection from cardiometabolic risk, even though associations between smoked tobacco and cardiometabolic risk are not captured by our model. This could be explained by its relatively low prevalence compared to smokeless tobacco (34.4% of current smokers, vs. 74.1% of smokeless users), because the cardiovascular risk is thought to be lower for smokeless that smoked tobacco [86].

Economic status on the other hand is inversely associated with smokeless tobacco use. The association of smokeless tobacco use with Yup’ik culture and inversely with economic status is suggestive of its role as a coping mechanism in Yup’ik culture [44]. The inverse association of smokeless tobacco with adiposity, but not with any of the other cardiometabolic risk components, could be interpreted as protective of cardiometabolic risk; however, iqmik, the main contributor to smokeless tobacco use for our participants, also has deleterious effects on health because of its high content in nicotine and multiple carcinogens [86,87]. The results show no other significant associations for economic status, including no direct significant relationship with processed foods. This is somewhat surprising as others have found associations with receiving food stamps or other government assistance and the consumption of processed foods [88,89].

Overall we did not find that increased education and economic status provided potential protective effects on cardiometabolic risk, in contrast with the health disparities reported in studies of non-Native populations [90,91], but consistent with findings in populations in early epidemiologic transition [92,93]. This may be due in part to the fact that there is less disparity in education and economic status in our population compared to other Arctic regions [37,94], which may impact access to traditional food sources in Alaska [95]. The Gini coefficient of income inequality in Alaska is the lowest of all states, at 0.41 vs. 0.47 nationwide; similarly, while proportions of people with 8^th^ grade education are similar in our study communities and the U.S. (7% and 5% respectively), the proportion of people with college education is much smaller in our study communities (13% vs. 56%) [96]. It is also possible that the communal aspect of the Yup’ik way of life reflected by the importance of community connections [63], where people in need may receive more support than in the general population. Such processes could moderate the deleterious effect of low income and education seen in non-Native studies or studies from other Arctic regions.

The associations of our Yup’ik Culture latent variable to smokeless tobacco and subsistence food consumption are not surprising since those behaviors can be considered part of Yup’ik culture. In addition, we found beneficial associations of Yup’ik culture participation and the TG-HDL lipids latent variable (HDL-C is reverse coded), adiposity, and blood pressure. Yup’ik culture participation has the largest beneficial association with adiposity of all model inputs, and adiposity serves as the key mediating variable for all of the health outcomes in the study. These results are compatible with the previous findings that Yup’ik people have lower prevalence of diabetes than the US average, despite a comparable rate of obesity [61,97,98]. We do not find an association between TG-HDL lipids and any of the dietary variables, possibly because we are evaluating a component combining HDL-C (reversed coded) and triglycerides, unlike other studies that looked at them individually. However, the path Yup’ik culture -> TG-HDL Lipids shows a substantial difference between direct and total effects (-0.06, ns direct, and 0.15 total, s), indicating the mediating role of adiposity. The inverse association of Yup’ik culture and TG-HDL lipids that we found was previously undescribed as such, although a strong inverse association between traditional food intake and TG, was described using a biomarker of traditional food intake (the δ^15^N value; [69] and FFQ dietary patterns [39]. The only potentially deleterious association of Yup’ik culture that we found is indirect, through its association with subsistence food consumption, itself associated with LDL lipids. The positive association of marine based traditional food with LDL-C had been previously described in our reference population, e.g., in [69], and is consistent with reported associations of LDL-C with ω-3 fatty acid supplementation [99], and of an experiment on the consumption of ω-3 rich fish associated with LDL-C [100]. We did not find the positive association of Yup’ik enculturation with physical activity reported by [42], possibly because we used different measures of physical activity and enculturation.

The association of Western culture with the consumption of processed foods and inverse association with the consumption of subsistence foods were expected as they had been reported previously [65]; in addition, we found an inverse association of Western cultural participation with blood pressure which may be in part explained by an increased level of health literacy [101] and could have implications for interventions by including a component aimed at increasing health literacy. We did not find an association of the consumption of subsistence foods with blood pressure similar to what was reported by Beaulieu-Jones et al. [102].

Importantly, we found indications of interaction effects between the cultural participation variables, indicating a potential effect of biculturalism on cardiometabolic health. The effects and interactions of differential cultural participation are unevenly distributed; for example, the interaction of Yup’ik and Western cultural participation shows that increasing participation in both potentially has important health benefits including lower LDL Lipids and lower blood pressure. Therefore, the beneficial health effects of following a Yup’ik way of life are most pronounced for those who identify with high levels of both Yup’ik and Western cultural practices. Interestingly, the fact that the association of Yup’ik culture with LDL lipids is in opposite direction for high and low levels of Western culture, represented by a significant interaction, explains why we do not find a significant main effect of Yup’ik culture on LDL lipids. The potentially beneficial role of biculturalism on blood pressure is similar to what was described before [46], but it is a new finding for LDL Lipids.

Finally, the results raise the question of whether and to what extent the associations to cardiometabolic risk factors are underwritten by both life course and gender roles. While female gender is associated with greater subsistence food use *and* adiposity, women show lower levels of both LDL lipids and blood pressure, despite adiposity’s overall positive association with both of these factors. A protective effect of estrogens on LDL lipids and blood pressure is possible [103]. Of note, gender on its own did not figure significantly in cultural participation for either Yup’ik or Western culture. Age, on the other hand, was associated with nearly all of the model components, including with Yup’ik culture and inversely with Western culture. The assumed implications of this pattern for food consumption hold. Increasing age (and thus greater adherence to a Yup’ik culture and lesser participation in Western culture) is also significantly associated with large increases in the consumption of subsistence foods, and a decreased consumption of processed foods. These same factors contributed to increases in adiposity, blood pressure and LDL lipids.

Clearly the role of social determinants of health is complex and understanding those complexities could be key to improving cardiometabolic health. Mixed economies in indigenous communities have featured elements of both traditional subsistence food harvests and consumption of commercially available mass-produced foods. While thought to be a transitional state that would bridge indigenous communities’ transition to a fully “modern” economy, combined food economies are now seen as a complex and ongoing set of food strategies that are unlikely to leave their “mixed” state at any time soon [37,53,95,104]. This points to the importance of preserving the subsistence activities embedded in Yup’ik culture participation from a community sustainability standpoint. Our results emphasize some of the potential health benefits of bicultural participation that may accompany this situation.

Other studies have also found varied impacts of acculturation on cardiometabolic health. For example, a study on the Inuit population in Greenland found that westernization was associated with the metabolic syndrome in men, attributed to a decrease of physical activity due to diminishing hunting and fishing, and an inverse association for women attributed to increase education [105]. A study in Hawaii found that people identifying highly with traditional, but not western culture, were more likely to have T2D [106]. A meta-analysis found that acculturation among Latinos in the United States had both a negative influence for certain health outcomes like substance abuse, dietary practices, and birth outcomes, but a positive one on health care use and access [107]. Another study found that Hispanics with lower levels of acculturation were more likely to have poorly controlled LDL cholesterol. The commonality of these results with ours suggests that health literacy is an important factor in the beneficial effect of Western culture. The divergence in the effects of adhering to the minority culture may be due in part to the fact that most studies, including ours, do not take into account the interaction between individuals and the social structure that they are embedded in, reflecting the fact that a relational approach to the agency-structure divide has not gained traction in health research [108]. Some novel frameworks, however, may help, such as substantialist approaches (modeling things rather than relations), a focus on cultural capital [21,109], or capital interplay [110,111].

Strengths of our study include the ability to combine socio-cultural, behavioral, and cardiometabolic risk factors in a single model; the use of an orthogonal model of culture, with both Yup’ik and Western latent variables; and the use of a novel methodology. Limitations include its observational and cross-sectional nature of the study, limiting causal interpretations, and the lack of a measure of stress (perceived or through biomarkers)—although our measure of economic status (running out of food for money or utilities) likely captured some element of stress. Limitations also include relatively limited measures of Yup’ik culture and economic status, a fitting of our outer model which is not perfect, difficulty of generalization even to other Alaska Native cultures, the inability to account for clustering at the community level in PLS-PM, a convenience sample, and the fact that we look at cardiometabolic risk factors rather than disease outcomes. Another important limitation is the choice of Yup’ik language use as a proxy for traditional cultural participation. While this strategy finds support in prior studies [112,113], it remains an indirect measure. In addition, this choice does not address questions of cultural consonance, or the extent to which individuals actually subscribe to identified cultural beliefs [114]. Cultural consonance has been shown to be an important consideration in indigenous health research [115], including work on cardiometabolic-related health [116]. The limitation that is most likely to have changed the interpretation of our results is the lack of measure of stress, since previous research points to the importance of stress both among our reference population [45], and as a determinant of cardiometabolic health in the general population [117]. A sensitivity analysis using multiple imputation rather than median values to replace the missing values, showed very little change in the results.

Overall, our analysis suggests that Yup’ik culture participation is an important determinant of cardiometabolic health in rural communities of southwestern Alaska. Bi-culturalism, demonstrated by the beneficial moderating effect of Western culture participation on the relationship between Yup’ik culture participation and blood pressure as well as LDL-cholesterol, was also significant. Although our analysis did not reproduce the potentially beneficial association between traditional food consumption and cardiometabolic risk factors found in previous studies, it uncovered significant potentially beneficial effects of Yup’ik culture participation on blood pressure, adiposity, and TG-HDL Lipids. Given the current understanding that mixed food economies are a permanent, rather than temporary, condition for many northern indigenous communities, such findings are important for long-term as well as short-term health planning. Our results suggest that reinforcing the protective effects of both Yup’ik *and* Western cultures could be useful in interventions, but new research involving Yup’ik collaborators, and harnessing knowledge gained from Yup’ik authors, e.g., [40,118], is needed to better understand the mechanisms underlying the effects of Yup’ik culture on cardiometabolic health. Such research would likely begin with questions of how stress can be mitigated by traditional practices and beliefs, and how health literacy may influence cardiometabolic health outcomes in bicultural settings.

## Supporting information

S1 Table Complete direct and total path coefficients for inner Partial Least Squares Path Model, with Bootstrap Standard Error and 95% confidence intervals (n = 486). (PDF)
