# Supplementary material for: Bi-cultural dynamics for risk and protective factors for cardiometabolic health in an Alaska Native (Yup’ik) population
Source: PLoS One. 2017 Nov 1;12(11):e0183451. doi: 10.1371/journal.pone.0183451 (PMC5665420; doi:10.1371/journal.pone.0183451)
Supplement: S1 Table — (PDF) [file pone.0183451.s001.pdf]

S1 Table. Complete direct and total path Coefficients for Inner Partial Least Squares Path Model, with Bootstrap Standard Error and 95% Confidence Intervals (n = 486).

Path	Direct Path				Total Effect			
	Coefficient	SE	CI Low	CI High	Coefficient	SE	CI Low	CI High
Gender -> Education	-0.005	0.043	-0.083	0.082	-0.005	0.043	-0.083	0.082
Gender -> Economic status	0.066	0.044	-0.026	0.146	0.066	0.044	-0.026	0.146
Gender -> Western culture	-0.010	0.044	-0.096	0.070	-0.010	0.044	-0.096	0.070
Gender -> Yup'ik culture	0.011	0.045	-0.072	0.097	0.011	0.045	-0.072	0.097
Gender -> Subsistence food	**0.168***	0.033	0.106	0.236	**0.175***	0.035	0.109	0.245
Gender -> Processed food	-0.001	0.043	-0.078	0.087	-0.002	0.045	-0.078	0.089
Gender -> Fruits and vegetables	0.074	0.047	-0.027	0.167	0.074	0.048	-0.022	0.172
Gender -> Physical activity	**-0.203***	0.043	-0.287	-0.119	**-0.202***	0.042	-0.285	-0.121
Gender -> Smoke tobacco	**-0.350***	0.039	-0.422	-0.273	**-0.350***	0.039	-0.422	-0.274
Gender -> Smokeless tobacco	**0.144***	0.045	0.057	0.236	**0.137***	0.046	0.048	0.227
Gender -> Adiposity	0.097	0.049	-0.001	0.192	**0.134***	0.044	0.052	0.220
Gender -> TG-HDL Lipids	**-0.293***	0.037	-0.368	-0.221	**-0.245***	0.037	-0.318	-0.174
Gender -> Glycemia	0.013	0.049	-0.094	0.105	0.038	0.045	-0.058	0.119
Gender -> Blood Pressure	**-0.295***	0.047	-0.380	-0.203	**-0.272***	0.044	-0.357	-0.182
Gender -> LDL Lipids	-0.052	0.042	-0.135	0.028	-0.003	0.040	-0.081	0.073
Age -> Education	-0.087	0.078	-0.215	0.079	-0.087	0.078	-0.215	0.079
Age -> Economic status	**-0.162***	0.049	-0.259	-0.070	**-0.169***	0.048	-0.264	-0.074
Age -> Western culture	**-0.216***	0.040	-0.294	-0.135	**-0.216***	0.040	-0.294	-0.135
Age -> Yup'ik culture	**0.318***	0.042	0.239	0.401	**0.318***	0.042	0.239	0.401
Age -> Subsistence food	**0.410***	0.040	0.337	0.489	**0.492***	0.036	0.422	0.564
Age -> Processed food	**-0.469***	0.040	-0.539	-0.380	**-0.513***	0.036	-0.574	-0.434
Age -> Fruits and vegetables	**-0.174***	0.048	-0.266	-0.081	**-0.198***	0.045	-0.285	-0.112
Age -> Physical activity	**-0.301***	0.048	-0.392	-0.201	**-0.320***	0.043	-0.397	-0.234
Age -> Smoke tobacco	**-0.167***	0.046	-0.255	-0.078	**-0.185***	0.040	-0.267	-0.110
Age -> Smokeless tobacco	0.049	0.051	-0.052	0.142	**0.116***	0.047	0.015	0.199
Age -> Adiposity	**0.224***	0.057	0.113	0.339	**0.202***	0.043	0.121	0.288
Age -> TG-HDL Lipids	**-0.422***	0.050	-0.513	-0.317	**-0.413***	0.037	-0.475	-0.333
Age -> Glycemia	**0.431***	0.073	0.306	0.547	**0.469***	0.058	0.375	0.539
Age -> Blood Pressure	**0.305***	0.059	0.188	0.426	**0.311***	0.041	0.230	0.391
Age -> LDL Lipids	**0.398***	0.055	0.285	0.500	**0.469***	0.036	0.402	0.539
Education -> Economic status	0.072	0.056	-0.034	0.185	0.072	0.056	-0.034	0.185
Education -> Subsistence food	0.063	0.046	-0.027	0.148	0.067	0.046	-0.024	0.153
Education -> Processed food	**0.130***	0.036	0.060	0.200	**0.131***	0.036	0.061	0.201
Education -> Fruits and vegetables	0.088	0.046	-0.005	0.176	0.090	0.045	-0.001	0.178
Education -> Physical activity	-0.018	0.038	-0.093	0.054	-0.016	0.038	-0.090	0.057

Path	Direct Path				Total Effect			
	Coefficient	SE	CI Low	CI High	Coefficient	SE	CI Low	CI High
Education -> Smoke tobacco	**-0.069***	0.036	-0.140	-0.001	**-0.069***	0.036	-0.138	0.000
Education -> Smokeless tobacco	-0.020	0.049	-0.121	0.072	-0.030	0.051	-0.130	0.070
Education -> Adiposity	0.075	0.040	-0.002	0.157	**0.100***	0.040	0.023	0.184
Education -> TG-HDL Lipids	0.026	0.041	-0.053	0.107	0.070	0.045	-0.016	0.160
Education -> Glycemia	-0.035	0.051	-0.125	0.074	-0.017	0.051	-0.106	0.088
Education -> Blood Pressure	-0.074	0.045	-0.164	0.013	-0.055	0.045	-0.146	0.035
Education -> LDL Lipids	0.096	0.049	-0.002	0.189	**0.124***	0.049	0.025	0.215
Economic status -> Subsistence food	0.052	0.038	-0.024	0.127	0.052	0.038	-0.024	0.127
Economic status -> Processed food	0.007	0.040	-0.068	0.089	0.007	0.040	-0.068	0.089
Economic status -> Fruits and vegetables	0.028	0.044	-0.061	0.116	0.028	0.044	-0.061	0.116
Economic status -> Physical activity	0.027	0.049	-0.067	0.122	0.027	0.049	-0.067	0.122
Economic status -> Smoke tobacco	0.003	0.042	-0.074	0.086	0.003	0.042	-0.074	0.086
Economic status -> Smokeless tobacco	**-0.136***	0.047	-0.225	-0.045	**-0.136***	0.047	-0.225	-0.045
Economic status -> Adiposity	-0.031	0.050	-0.127	0.068	-0.015	0.050	-0.111	0.084
Economic status -> TG-HDL Lipids	0.022	0.037	-0.057	0.093	0.015	0.043	-0.072	0.097
Economic status -> Glycemia	0.058	0.043	-0.033	0.136	0.058	0.045	-0.034	0.140
Economic status -> Blood Pressure	-0.052	0.042	-0.129	0.028	-0.056	0.044	-0.135	0.027
Economic status -> LDL Lipids	-0.015	0.045	-0.100	0.074	-0.002	0.047	-0.088	0.093
Western culture -> Subsistence food	**-0.175***	0.049	-0.260	-0.077	**-0.175***	0.049	-0.260	-0.077
Western culture -> Processed food	**0.185***	0.055	0.066	0.289	**0.185***	0.055	0.066	0.289
Western culture -> Fruits and vegetables	0.119	0.066	-0.007	0.253	0.119	0.066	-0.007	0.253
Western culture -> Physical activity	0.090	0.064	-0.039	0.216	0.090	0.064	-0.039	0.216
Western culture -> Smoke tobacco	0.052	0.071	-0.084	0.191	0.052	0.071	-0.084	0.191
Western culture -> Smokeless tobacco	0.055	0.066	-0.075	0.184	0.055	0.066	-0.075	0.184
Western culture -> Adiposity	-0.072	0.068	-0.201	0.058	-0.084	0.067	-0.212	0.050
Western culture -> TG-HDL Lipids	-0.006	0.054	-0.107	0.104	-0.025	0.056	-0.132	0.088
Western culture -> Glycemia	-0.027	0.058	-0.140	0.080	-0.052	0.057	-0.166	0.055
Western culture -> Blood Pressure	**-0.113***	0.060	-0.231	-0.004	**-0.120***	0.062	-0.241	-0.001
Western culture -> LDL Lipids	-0.033	0.064	-0.150	0.091	-0.075	0.065	-0.192	0.055
Yup'ik culture -> Subsistence food	**0.185***	0.055	0.081	0.294	**0.185***	0.055	0.081	0.294
Yup'ik culture -> Processed food	0.027	0.055	-0.092	0.128	0.027	0.055	-0.092	0.128
Yup'ik culture -> Fruits and vegetables	0.044	0.074	-0.098	0.188	0.044	0.074	-0.098	0.188
Yup'ik culture -> Physical activity	0.010	0.065	-0.124	0.120	0.010	0.065	-0.124	0.120
Yup'ik culture -> Smoke tobacco	-0.039	0.073	-0.188	0.106	-0.039	0.073	-0.188	0.106
Yup'ik culture -> Smokeless tobacco	**0.170***	0.077	0.030	0.322	**0.170***	0.077	0.030	0.322
Yup'ik culture -> Adiposity	**-0.189***	0.079	-0.333	-0.023	**-0.188***	0.079	-0.335	-0.026
Yup'ik culture -> TG-HDL Lipids	-0.057	0.050	-0.156	0.038	**-0.145***	0.057	-0.256	-0.036
Yup'ik culture -> Glycemia	-0.029	0.060	-0.155	0.077	-0.061	0.060	-0.188	0.044
Yup'ik culture -> Blood Pressure	**-0.153***	0.062	-0.275	-0.026	**-0.205***	0.066	-0.332	-0.075

Path	Direct Path				Total Effect			
	Coefficient	SE	CI Low	CI High	Coefficient	SE	CI Low	CI High
Yup'ik culture -> LDL Lipids	-0.096	0.071	-0.236	0.047	-0.105	0.073	-0.243	0.036
Subsistence food -> Adiposity	0.078	0.055	-0.023	0.185	0.078	0.055	-0.023	0.185
Subsistence food -> TG-HDL Lipids	-0.060	0.049	-0.160	0.031	-0.030	0.053	-0.136	0.074
Subsistence food -> Glycemia	0.017	0.062	-0.102	0.141	0.030	0.064	-0.090	0.161
Subsistence food -> Blood Pressure	-0.054	0.055	-0.162	0.049	-0.035	0.056	-0.151	0.068
Subsistence food -> LDL Lipids	**0.147***	0.053	0.046	0.248	**0.159***	0.053	0.056	0.259
Processed food -> Adiposity	0.081	0.066	-0.037	0.222	0.081	0.066	-0.037	0.222
Processed food -> TG-HDL Lipids	0.062	0.049	-0.023	0.158	0.094	0.058	-0.004	0.215
Processed food -> Glycemia	-0.017	0.055	-0.124	0.083	-0.003	0.054	-0.113	0.099
Processed food -> Blood Pressure	-0.015	0.058	-0.120	0.109	0.004	0.060	-0.099	0.131
Processed food -> LDL Lipids	-0.011	0.055	-0.124	0.089	0.002	0.054	-0.108	0.102
Fruits and vegetables -> Adiposity	0.046	0.057	-0.068	0.158	0.046	0.057	-0.068	0.158
Fruits and vegetables -> TG-HDL Lipids	-0.034	0.043	-0.121	0.048	-0.016	0.048	-0.114	0.076
Fruits and vegetables -> Glycemia	-0.054	0.050	-0.155	0.045	-0.046	0.050	-0.142	0.058
Fruits and vegetables -> Blood Pressure	0.039	0.045	-0.051	0.130	0.049	0.049	-0.047	0.148
Fruits and vegetables -> LDL Lipids	0.002	0.046	-0.086	0.090	0.009	0.046	-0.076	0.099
Physical activity -> Adiposity	**-0.133***	0.041	-0.211	-0.050	**-0.133***	0.041	-0.211	-0.050
Physical activity -> TG-HDL Lipids	-0.024	0.039	-0.107	0.049	-0.077	0.043	-0.168	0.002
Physical activity -> Glycemia	0.056	0.047	-0.038	0.144	0.033	0.046	-0.058	0.119
Physical activity -> Blood Pressure	0.028	0.044	-0.059	0.112	-0.003	0.045	-0.090	0.085
Physical activity -> LDL Lipids	0.041	0.041	-0.039	0.124	0.020	0.040	-0.061	0.095
Smoke tobacco -> Adiposity	-0.031	0.051	-0.133	0.064	-0.031	0.051	-0.133	0.064
Smoke tobacco -> TG-HDL Lipids	-0.015	0.044	-0.109	0.064	-0.027	0.049	-0.126	0.061
Smoke tobacco -> Glycemia	-0.038	0.046	-0.119	0.064	-0.043	0.046	-0.124	0.059
Smoke tobacco -> Blood Pressure	-0.024	0.046	-0.111	0.064	-0.031	0.048	-0.128	0.056
Smoke tobacco -> LDL Lipids	-0.056	0.047	-0.149	0.032	-0.061	0.046	-0.155	0.026
Smokeless tobacco -> Adiposity	**-0.103***	0.044	-0.187	-0.018	**-0.103***	0.044	-0.187	-0.018
Smokeless tobacco -> TG-HDL Lipids	-0.021	0.042	-0.100	0.059	-0.061	0.047	-0.147	0.028
Smokeless tobacco -> Glycemia	-0.018	0.047	-0.117	0.064	-0.035	0.047	-0.131	0.050
Smokeless tobacco -> Blood Pressure	-0.006	0.046	-0.096	0.086	-0.030	0.046	-0.118	0.061
Smokeless tobacco -> LDL Lipids	-0.049	0.045	-0.139	0.038	-0.065	0.045	-0.152	0.024
Adiposity -> TG-HDL Lipids	**0.393***	0.036	0.323	0.465	**0.393***	0.036	0.323	0.465
Adiposity -> Glycemia	**0.169***	0.052	0.075	0.275	**0.169***	0.052	0.075	0.275
Adiposity -> Blood Pressure	**0.232***	0.052	0.130	0.330	**0.232***	0.052	0.130	0.330
Adiposity -> LDL Lipids	**0.159***	0.051	0.066	0.268	**0.159***	0.051	0.066	0.268
Gender -> Education	-0.005	0.043	-0.083	0.082	-0.005	0.043	-0.083	0.082
Gender -> Economic status	0.066	0.044	-0.026	0.146	0.066	0.044	-0.026	0.146
Gender -> Western culture	-0.010	0.044	-0.096	0.070	-0.010	0.044	-0.096	0.070
Gender -> Yup'ik culture	0.011	0.045	-0.072	0.097	0.011	0.045	-0.072	0.097

Path	Direct Path				Total Effect			
	Coefficient	SE	CI Low	CI High	Coefficient	SE	CI Low	CI High
Gender -> Subsistence food	**0.168***	0.033	0.106	0.236	**0.175***	0.035	0.109	0.245
Gender -> Processed food	-0.001	0.043	-0.078	0.087	-0.002	0.045	-0.078	0.089
Gender -> Fruits and vegetables	0.074	0.047	-0.027	0.167	0.074	0.048	-0.022	0.172
Gender -> Physical activity	**-0.203***	0.043	-0.287	-0.119	**-0.202***	0.042	-0.285	-0.121
Gender -> Smoke tobacco	**-0.350***	0.039	-0.422	-0.273	**-0.350***	0.039	-0.422	-0.274
Gender -> Smokeless tobacco	**0.144***	0.045	0.057	0.236	**0.137***	0.046	0.048	0.227
Gender -> Adiposity	0.097	0.049	-0.001	0.192	**0.134***	0.044	0.052	0.220
Gender -> TG-HDL Lipids	**-0.293***	0.037	-0.368	-0.221	**-0.245***	0.037	-0.318	-0.174
Gender -> Glycemia	0.013	0.049	-0.094	0.105	0.038	0.045	-0.058	0.119
Gender -> Blood Pressure	**-0.295***	0.047	-0.380	-0.203	**-0.272***	0.044	-0.357	-0.182
Gender -> LDL Lipids	-0.052	0.042	-0.135	0.028	-0.003	0.040	-0.081	0.073
Age -> Education	-0.087	0.078	-0.215	0.079	-0.087	0.078	-0.215	0.079
Age -> Economic status	**-0.162***	0.049	-0.259	-0.070	**-0.169***	0.048	-0.264	-0.074
Age -> Western culture	**-0.216***	0.040	-0.294	-0.135	**-0.216***	0.040	-0.294	-0.135
Age -> Yup’ik culture	**0.318***	0.042	0.239	0.401	**0.318***	0.042	0.239	0.401
Age -> Subsistence food	**0.410***	0.040	0.337	0.489	**0.492***	0.036	0.422	0.564
Age -> Processed food	**-0.469***	0.040	-0.539	-0.380	**-0.513***	0.036	-0.574	-0.434
Age -> Fruits and vegetables	**-0.174***	0.048	-0.266	-0.081	**-0.198***	0.045	-0.285	-0.112
Age -> Physical activity	**-0.301***	0.048	-0.392	-0.201	**-0.320***	0.043	-0.397	-0.234
Age -> Smoke tobacco	**-0.167***	0.046	-0.255	-0.078	**-0.185***	0.040	-0.267	-0.110
Age -> Smokeless tobacco	0.049	0.051	-0.052	0.142	**0.116***	0.047	0.015	0.199
Age -> Adiposity	**0.224***	0.057	0.113	0.339	**0.202***	0.043	0.121	0.288
Age -> TG-HDL Lipids	**-0.422***	0.050	-0.513	-0.317	**-0.413***	0.037	-0.475	-0.333
Age -> Glycemia	**0.431***	0.073	0.306	0.547	**0.469***	0.058	0.375	0.539
Age -> Blood Pressure	**0.305***	0.059	0.188	0.426	**0.311***	0.041	0.230	0.391
Age -> LDL Lipids	**0.398***	0.055	0.285	0.500	**0.469***	0.036	0.402	0.539
Education -> Economic status	0.072	0.056	-0.034	0.185	0.072	0.056	-0.034	0.185
Education -> Subsistence food	0.063	0.046	-0.027	0.148	0.067	0.046	-0.024	0.153
Education -> Processed food	**0.130***	0.036	0.060	0.200	**0.131***	0.036	0.061	0.201
Education -> Fruits and vegetables	0.088	0.046	-0.005	0.176	0.090	0.045	-0.001	0.178
Education -> Physical activity	-0.018	0.038	-0.093	0.054	-0.016	0.038	-0.090	0.057
Education -> Smoke tobacco	**-0.069***	0.036	-0.140	-0.001	**-0.069***	0.036	-0.138	0.000
Education -> Smokeless tobacco	-0.020	0.049	-0.121	0.072	-0.030	0.051	-0.130	0.070
Education -> Adiposity	0.075	0.040	-0.002	0.157	**0.100***	0.040	0.023	0.184
Education -> TG-HDL Lipids	0.026	0.041	-0.053	0.107	0.070	0.045	-0.016	0.160
Education -> Glycemia	-0.035	0.051	-0.125	0.074	-0.017	0.051	-0.106	0.088
Education -> Blood Pressure	-0.074	0.045	-0.164	0.013	-0.055	0.045	-0.146	0.035
Education -> LDL Lipids	0.096	0.049	-0.002	0.189	**0.124***	0.049	0.025	0.215
Economic status -> Subsistence food	0.052	0.038	-0.024	0.127	0.052	0.038	-0.024	0.127

Path	Direct Path				Total Effect			
	**Coefficient**	**SE**	**CI Low**	**CI High**	**Coefficient**	**SE**	**CI Low**	**CI High**
Economic status -> Processed food	0.007	0.040	-0.068	0.089	0.007	0.040	-0.068	0.089
Economic status -> Fruits and vegetables	0.028	0.044	-0.061	0.116	0.028	0.044	-0.061	0.116
Economic status -> Physical activity	0.027	0.049	-0.067	0.122	0.027	0.049	-0.067	0.122
Economic status -> Smoke tobacco	0.003	0.042	-0.074	0.086	0.003	0.042	-0.074	0.086
Economic status -> Smokeless tobacco	**-0.136***	0.047	-0.225	-0.045	**-0.136***	0.047	-0.225	-0.045
Economic status -> Adiposity	-0.031	0.050	-0.127	0.068	-0.015	0.050	-0.111	0.084
Economic status -> TG-HDL Lipids	0.022	0.037	-0.057	0.093	0.015	0.043	-0.072	0.097
Economic status -> Glycemia	0.058	0.043	-0.033	0.136	0.058	0.045	-0.034	0.140
Economic status -> Blood Pressure	-0.052	0.042	-0.129	0.028	-0.056	0.044	-0.135	0.027
Economic status -> LDL Lipids	-0.015	0.045	-0.100	0.074	-0.002	0.047	-0.088	0.093
Western culture -> Subsistence food	**-0.175***	0.049	-0.260	-0.077	**-0.175***	0.049	-0.260	-0.077
Western culture -> Processed food	**0.185***	0.055	0.066	0.289	**0.185***	0.055	0.066	0.289
Western culture -> Fruits and vegetables	0.119	0.066	-0.007	0.253	0.119	0.066	-0.007	0.253
Western culture -> Physical activity	0.090	0.064	-0.039	0.216	0.090	0.064	-0.039	0.216
Western culture -> Smoke tobacco	0.052	0.071	-0.084	0.191	0.052	0.071	-0.084	0.191
Western culture -> Smokeless tobacco	0.055	0.066	-0.075	0.184	0.055	0.066	-0.075	0.184
Western culture -> Adiposity	-0.072	0.068	-0.201	0.058	-0.084	0.067	-0.212	0.050
Western culture -> TG-HDL Lipids	-0.006	0.054	-0.107	0.104	-0.025	0.056	-0.132	0.088
Western culture -> Glycemia	-0.027	0.058	-0.140	0.080	-0.052	0.057	-0.166	0.055
Western culture -> Blood Pressure	**-0.113***	0.060	-0.231	-0.004	**-0.120***	0.062	-0.241	-0.001
Western culture -> LDL Lipids	-0.033	0.064	-0.150	0.091	-0.075	0.065	-0.192	0.055
Yup'ik culture -> Subsistence food	**0.185***	0.055	0.081	0.294	**0.185***	0.055	0.081	0.294
Yup'ik culture -> Processed food	0.027	0.055	-0.092	0.128	0.027	0.055	-0.092	0.128
Yup'ik culture -> Fruits and vegetables	0.044	0.074	-0.098	0.188	0.044	0.074	-0.098	0.188
Yup'ik culture -> Physical activity	0.010	0.065	-0.124	0.120	0.010	0.065	-0.124	0.120
Yup'ik culture -> Smoke tobacco	-0.039	0.073	-0.188	0.106	-0.039	0.073	-0.188	0.106
Yup'ik culture -> Smokeless tobacco	**0.170***	0.077	0.030	0.322	**0.170***	0.077	0.030	0.322
Yup'ik culture -> Adiposity	**-0.189***	0.079	-0.333	-0.023	**-0.188***	0.079	-0.335	-0.026
Yup'ik culture -> TG-HDL Lipids	-0.057	0.050	-0.156	0.038	**-0.145***	0.057	-0.256	-0.036
Yup'ik culture -> Glycemia	-0.029	0.060	-0.155	0.077	-0.061	0.060	-0.188	0.044
Yup'ik culture -> Blood Pressure	**-0.153***	0.062	-0.275	-0.026	**-0.205***	0.066	-0.332	-0.075
Yup'ik culture -> LDL Lipids	-0.096	0.071	-0.236	0.047	-0.105	0.073	-0.243	0.036
Subsistence food -> Adiposity	0.078	0.055	-0.023	0.185	0.078	0.055	-0.023	0.185
Subsistence food -> TG-HDL Lipids	-0.060	0.049	-0.160	0.031	-0.030	0.053	-0.136	0.074
Subsistence food -> Glycemia	0.017	0.062	-0.102	0.141	0.030	0.064	-0.090	0.161
Subsistence food -> Blood Pressure	-0.054	0.055	-0.162	0.049	-0.035	0.056	-0.151	0.068
Subsistence food -> LDL Lipids	**0.147***	0.053	0.046	0.248	**0.159***	0.053	0.056	0.259
Processed food -> Adiposity	0.081	0.066	-0.037	0.222	0.081	0.066	-0.037	0.222
Processed food -> TG-HDL Lipids	0.062	0.049	-0.023	0.158	0.094	0.058	-0.004	0.215

Path	Direct Path				Total Effect			
	**Coefficient**	**SE**	**CI Low**	**CI High**	**Coefficient**	**SE**	**CI Low**	**CI High**
Processed food -> Glycemia	-0.017	0.055	-0.124	0.083	-0.003	0.054	-0.113	0.099
Processed food -> Blood Pressure	-0.015	0.058	-0.120	0.109	0.004	0.060	-0.099	0.131
Processed food -> LDL Lipids	-0.011	0.055	-0.124	0.089	0.002	0.054	-0.108	0.102
Fruits and vegetables -> Adiposity	0.046	0.057	-0.068	0.158	0.046	0.057	-0.068	0.158
Fruits and vegetables -> TG-HDL Lipids	-0.034	0.043	-0.121	0.048	-0.016	0.048	-0.114	0.076
Fruits and vegetables -> Glycemia	-0.054	0.050	-0.155	0.045	-0.046	0.050	-0.142	0.058
Fruits and vegetables -> Blood Pressure	0.039	0.045	-0.051	0.130	0.049	0.049	-0.047	0.148
Fruits and vegetables -> LDL Lipids	0.002	0.046	-0.086	0.090	0.009	0.046	-0.076	0.099
Physical activity -> Adiposity	**-0.133***	0.041	-0.211	-0.050	**-0.133***	0.041	-0.211	-0.050
Physical activity -> TG-HDL Lipids	-0.024	0.039	-0.107	0.049	-0.077	0.043	-0.168	0.002
Physical activity -> Glycemia	0.056	0.047	-0.038	0.144	0.033	0.046	-0.058	0.119
Physical activity -> Blood Pressure	0.028	0.044	-0.059	0.112	-0.003	0.045	-0.090	0.085
Physical activity -> LDL Lipids	0.041	0.041	-0.039	0.124	0.020	0.040	-0.061	0.095
Smoke tobacco -> Adiposity	-0.031	0.051	-0.133	0.064	-0.031	0.051	-0.133	0.064
Smoke tobacco -> TG-HDL Lipids	-0.015	0.044	-0.109	0.064	-0.027	0.049	-0.126	0.061
Smoke tobacco -> Glycemia	-0.038	0.046	-0.119	0.064	-0.043	0.046	-0.124	0.059
Smoke tobacco -> Blood Pressure	-0.024	0.046	-0.111	0.064	-0.031	0.048	-0.128	0.056
Smoke tobacco -> LDL Lipids	-0.056	0.047	-0.149	0.032	-0.061	0.046	-0.155	0.026
Smokeless tobacco -> Adiposity	**-0.103***	0.044	-0.187	-0.018	**-0.103***	0.044	-0.187	-0.018
Smokeless tobacco -> TG-HDL Lipids	-0.021	0.042	-0.100	0.059	-0.061	0.047	-0.147	0.028
Smokeless tobacco -> Glycemia	-0.018	0.047	-0.117	0.064	-0.035	0.047	-0.131	0.050
Smokeless tobacco -> Blood Pressure	-0.006	0.046	-0.096	0.086	-0.030	0.046	-0.118	0.061
Smokeless tobacco -> LDL Lipids	-0.049	0.045	-0.139	0.038	-0.065	0.045	-0.152	0.024
Adiposity -> TG-HDL Lipids	**0.393***	0.036	0.323	0.465	**0.393***	0.036	0.323	0.465
Adiposity -> Glycemia	**0.169***	0.052	0.075	0.275	**0.169***	0.052	0.075	0.275
Adiposity -> Blood Pressure	**0.232***	0.052	0.130	0.330	**0.232***	0.052	0.130	0.330
Adiposity -> LDL Lipids	**0.159***	0.051	0.066	0.268	**0.159***	0.051	0.066	0.268

* Significant effects (α=0.05)

Notes: Goodness of fit (GoF) for model is 0.35.Based on cutoff values for Cohen f^2 of .02, .15, and .35, our *f2* values indicate high effects sizes for TG-HDL Lipids = 0.68, Subsistence food = 0.63, Processed food = 0.45, LDL Lipids = 0.39, Glycemia = 0.35, medium for Blood Pressure = 0.32, Smoke tobacco = 0.21, Physical activity = 0.18, and small for Adiposity = 0.14, Yup'ik culture = 0.11, Smokeless tobacco = 0.08, Fruits and vegetables = 0.07, Western culture = 0.05, and Economic status = 0.04.
